# Epidemiological transitions in human evolution and the richness of viruses, helminths, and protozoa

**DOI:** 10.1093/emph/eoab009

**Published:** 2021-02-23

**Authors:** Caroline R Amoroso, Charles L Nunn

**Affiliations:** 1 Department of Evolutionary Anthropology, Duke University, 130 Science Dr, Durham, NC 27707, USA; 2 Department of Biology, University of Virginia, 485 McCormick Rd, Charlottesville, VA 22904, USA; 3 Duke Global Health Institute, Duke University, 310 Trent Dr, Durham, NC 27710, USA

**Keywords:** phylogenetic comparative analysis, disease, host-pathogen interactions, parasite diversity, species richness, human uniqueness

## Abstract

**Background and objectives:**

In absolute terms, humans are extremely highly parasitized compared to other primates. This may reflect that humans are outliers in traits correlated with parasite richness: population density, geographic range area, and study effort. The high degree of parasitism could also reflect amplified disease risk associated with agriculture and urbanization. Alternatively, controlling for other variables, cultural and psychological adaptations could have reduced parasitism in humans over evolutionary time.

**Methodology:**

We predicted the number of parasites that would infect a nonhuman primate with human phenotypic characteristics and phylogenetic position, and then compared observed parasitism of humans in eight geopolitical countries to the predicted distributions. The analyses incorporated study effort, phylogeny, and drivers of parasitism in 33 primate species.

**Results:**

Analyses of individual countries were not supportive of either hypothesis. When analyzed collectively, however, human populations showed consistently lower than expected richness of protozoa and helminths, but higher richness of viruses. Thus, human evolutionary innovations and new parasite exposures may have impacted groups of parasites in different ways, with support for both hypotheses in the overall analysis.

**Conclusions and implications:**

The high level of parasitism observed in humans only applies to viruses, and was not extreme in any of our tests of individual countries. In contrast, we find consistent reductions in protozoa and helminths across countries, suggesting reduced parasitism by these groups during human evolution. We propose that hygienic and technological advances might have extinguished fecal-orally or indirectly transmitted parasites like helminths, whereas higher human densities and host-shifting potential of viruses have supported increased virus richness.

**Lay Summary:**

Vastly more parasite species infect humans than any other primate host. Controlling for factors that influence parasite richness, such as the intensity of study effort and body mass, we find that humans may have more viruses, but fewer helminths and protozoa, than expected based on evolutionary analyses of parasitism in other primates.

## INTRODUCTION

Humans have undergone a series of epidemiological transitions that have changed the composition and richness of their parasites [1]. New zoonotic infections emerged when humans shifted from a hunting-and-gathering mode of subsistence to a more settled, agriculture-based lifestyle that included close contact with domesticated animals [2, 3]. Later, the aggregation of people into urban centers led to the emergence of “crowd” diseases like measles and smallpox, which could persist only after human populations reached a critical size [3]. Permanent settlement, agriculture, close contact with domesticated animals, and crowding are human traits commonly used to explain the exceptionally high diversity of human parasites and pathogens, which stands at 1415 species by one count [2] and 2107 by another count [4]. By comparison, our closest great ape relatives all have fewer than 100 parasites recorded [5, 6], and share fewer than expected parasites with humans [4, 7].

Thus, at first glance, it appears that humans are hyper-parasitized, and it is tempting to point to key transitions in human evolution, including agriculture and the rise of highly dense urban populations, as the cause. Several factors, however, call into question this view of human exceptionalism. One factor involves the intensity of study effort on the parasites of primate hosts: humans are much more intensively studied for infectious organisms than are other primates, leading to a greater discovery of parasites and pathogens. This aspect of sampling bias affects many investigations of species richness, including studies of both parasites and free-living organisms [8–11]. In addition, humans have relatively large body mass among the primates. Larger bodies could provide more niches for parasites to colonize, and body mass covaries with increased exposure through greater food intake [8, 12]. Another factor is geographic range size. Humans have successfully colonized most terrestrial landmasses on Earth, with a geographic range size that is at least an order of magnitude larger than the range of any other primate. Parasite richness covaries with geographic range size because a host with a larger geographic range is likely to experience more diverse habitats across its range, resulting in contact with more different parasites and pathogens [13]. Thus, it could be that exceptional human parasitism simply reflects the extensive geographic range of the human species, especially when combined with greater study effort, higher population density, and relatively large body size.

Another factor could plausibly have the opposite effect—to reduce human parasite richness—but it is rarely considered. Humans possess a multitude of cultural behaviors for avoiding parasites [14]. One example is cooking, a uniquely human behavior that increases the available energy in food [15] and also reduces the risk of exposure to parasites in food [16]. Similarly, use of spices in cooked foods may play a role in preventing spoilage [17]. In addition to the example of medical treatment that may come to mind, humans practice many diverse hygienic behaviors [18], such as washing hands before eating or using only one hand for unsanitary practices, and avoid potentially infectious materials, motivated by the emotion of disgust [19, 20]. While infection avoidance behaviors are present in other animals [21–23], the extensive, pervasive, and culturally perpetuated nature of the disgust response across human societies may be a feature that distinguishes humans from other animals [24]. These innovations and their social transmission set humans apart from other animal species, and thus could account for a reduction in parasite exposure across humans’ evolutionary history.

Here, we investigate the hypothesis that humans are hyper-parasitized based on our epidemiological transitions (the *hyper-parasitism hypothesis*), versus the alternative that cultural adaptations have contributed to lower parasitism in humans than nonhuman primates (the *parasite reduction hypothesis*). We simultaneously control for study effort, geographic range, phylogeny, and other variables that influence total parasite counts in a host species. The parasites of humans and nonhuman primates are diverse, spanning a variety of taxonomic groups, transmission modes, mutation rates, and life cycles [8, 25]. Given this diversity, we expect that different groups of parasites could follow different patterns of evolution or extinction across the various epidemiological transitions of their human host. We examine results on the level of individual human populations to assess the strength of deviations from expectations, and across human populations to assess overall consistency of the effects.

## METHODOLOGY

### Data collection

#### Nonhuman primate data

We used published data on the parasites of nonhuman primates to generate predictions for human parasitism. Data on parasite species richness for the nonhuman primates were obtained from the Global Mammal Parasite Database (database accessed on June 5, 2019) [5, 6]. We focused on parasites classified as helminths, protozoa, and viruses, which are the best-studied groups of parasites infecting primates. We included nonhuman primate species for which at least two species of helminths (including cestodes, nematodes, and trematodes), protozoa, and viruses had each been described in at least two papers in the literature, thus limiting our study to 33 primate host species. Using data from the database, we constructed a matrix of parasite species as reported by each original study in the database for each host species, which was used to control for study effort using a richness estimator from ecology (see below).

The nonhuman primate trait data were previously published [8], with a missing data point for population density of *Mandrillus sphinx* obtained from the literature [26]. A posterior distribution of 100 phylogenetic trees was downloaded from the 10KTrees Website [27] and used to account for phylogenetic uncertainty when conducting the tree-based comparative analyses.

#### Human data

A major challenge of comparing humans and nonhuman primates is the scale of variation: some of the relevant ecological and biogeographical traits used as predictors in our model (such as geographic range and latitudinal range) differ by orders of magnitude between human and nonhuman primates. To avoid extrapolating our model predictions for humans beyond the scope of the nonhuman primate data, we chose to study eight country-level populations of humans on three continents where nonhuman primates also live: Bolivia, Colombia, Kenya, India, Indonesia, Madagascar, Nigeria, and Vietnam. As with the nonhuman primates, we collected richness data on three parasite types: helminths, protozoa, and viruses.

The human data were extracted from the Global Infectious Disease and Epidemiology Online Network (GIDEON; https://www.gideononline.com/), a resource for medical doctors that organizes infectious disease information by country. Data were extracted from GIDEON for the eight countries and three parasite types of interest (database accessed between September 10, 2015 and June 30, 2016).

We reviewed the list of references in GIDEON for each disease in each country that was caused by a parasite from one of the three groups included in this study (helminths, protozoa, and viruses). We removed any duplicate listings from the reference list and read the title and abstract of each reference to determine the nature of the study. We included studies based on whether the sampling methods were judged to be comparable to those typically used to sample nonhuman primates. Specifically, we included studies that, based on their title and abstract, could be judged to present original data that reported on the presence of a particular parasite, or a parasite whose identity could be reasonably inferred from the context of the title and abstract, as determined by samples collected directly from human subjects. Examples of references that we included are as follows: prevalence surveys of a population, accounts of particular incidences or case studies, or surveys of patients at a hospital presenting with certain symptoms (e.g., prevalence of hookworm in patients with diarrhea). We excluded reviews, perspectives, recommendations, or analyses of previously published data. We also excluded reports from within immuno-compromised patients, reports that only included symptoms, and proMed emails and other outbreak reports that were not published in a journal. We did not include studies or surveys of the disease in nonhuman host species, nor studies that surveyed for the parasite in the environment. If no parasite species was named in the title or abstract, we assigned the paper to the parasite associated with the disease in which the paper was categorized in the database. We inferred species counts from the reported diseases as conservatively as possible; for example, if multiple papers reported “Adenovirus,” we counted that as one species in that country unless the studies identified multiple lineages of Adenovirus to the species level. From this list of studies and parasite species reported in the studies, we created a matrix of studies by parasite species, which we used to control for study effort.

Geographic range size for the eight human populations was the area of each country as reported by the CIA World Factbook, and population densities for the eight countries of interest were calculated using the corresponding values for population and geographic area of each country [28]. We used body mass estimates for continental regions [29]. Latitudinal ranges of the countries were obtained based on the maximum estimated northern and southern extent using Google Earth.

The data that were analyzed in this study are available as Supplementary Materials.

#### Controlling for study effort

A major logistical concern when comparing the parasite richness of humans and nonhuman primates is that the intensity of sampling for parasites varies across and within host species, and the relationship between study effort and parasite species richness is often nonlinear, especially within well-sampled species. To address this problem, we calculated Chao2 estimates [10] for the parasite richness of each parasite type for each host. The Chao2 method estimates species richness from patterns of incidence across sampling events [30]. Based on analysis of artificial datasets simulated to mimic the structure of the Global Mammal Parasite Database, we previously found that estimating richness using Chao2 is relatively more precise and unbiased than other methods of controlling for uneven sampling effort across species, and especially useful for making comparisons across species [11]. In this study, we thus use an estimate of species richness that is based on patterns of parasite species discovery in different studies, rather than an observed value (although we refer to this value as “observed” to draw a contrast with the predicted values, see below). To estimate Chao2, we used the “fossil” package for R version 3.6.0 [31,32]. All data were log_10_ transformed prior to analysis.

### Phylogenetic prediction: effects within human populations (countries)

For each parasite type, we used BayesModelS, which employs a Bayesian Markov Chain Monte Carlo (MCMC) method to fit a phylogenetic least-squares regression model [33, 34]. The model also estimates λ, which scales the phylogeny based on the degree of phylogenetic signal in the model residuals. The parameter λ ranges from 0 when there is no phylogenetic signal to 1 when the provided phylogeny accounts for phylogenetic signal under a Brownian motion model of evolution [35].

We fit models for each parasite type to the nonhuman primate data, with Chao2-based estimate for parasite richness (*PSR*, hereafter, “observed richness”) as the response, and body mass (*BodyMass*), geographic range area (*GeoRange*), latitudinal range (*LatRange*), and population density (*PopDens*) as predictors (Supplementary Information S1). The models thus took the following form,

$$PSR_{i}\;∼\;\mathrm{BodyMass}+\mathrm{GeoRange}+\mathrm{LatRa}nge+\mathrm{PopDens}+\;\varepsilon$$

where i represents the parasite type (helminths, protozoa, or viruses), and ε represents the phylogenetic covariance structure of the data. With a posterior distribution of coefficients from this model, including λ and the intercept, we then used estimated values of the predictors from humans to generate posterior predictions of human parasite richness for each of the eight human populations. We ran 210 000 iterations for each model with a burn-in of 10 000, and we thinned the output by 100 to reduce autocorrelation between runs. This resulted in a posterior distribution of 2 000 predicted richness values for each parasite type in each human country. We generated predicted distributions of parasite richness of helminths, protozoa, and viruses separately for each of the human populations (24 posterior distributions of predictions). When we compared the observed richness to the prediction generated by the nonhuman primate model, we considered exceptional any observed human parasite richness that was more extreme (either higher or lower) than 5% of the posterior distribution: i.e., outside of the 90% credible interval. We also compared whether the mean of the posterior distribution for the predicted value was greater or less than the observed value. As a diagnostic, we also used the models to predict parasitism for each nonhuman primate species and compared the observed values of Chao2-based PSR to those predictions (Supplementary Information S2). The model performed well by this metric, with no consistent biases in the predictions of the nonhuman primate parasitism.

### Consistency: Predictors of deviations from expectations across populations

To assess whether deviations of the observed richness from predictions were consistent across countries, we modeled the difference in observed richness from the mean of the posterior predicted distribution from the phylogenetic prediction analysis. The mean was an appropriate measure of central tendency because the posterior distributions were generally symmetric around the mean. We then used a general linear model to investigate this deviation, with parasite group as a predictor. With three parasite groups (helminths, protozoa, and viruses) and 8 countries, this analysis involved the output from 24 separate tests. As with the other analyses, we used a Bayesian approach to fit this model, with the rethinking [36] and rstan [37] packages in R. For this, we fit an MCMC model with four chains and 5 000 iterations. To assess effects, we examined 90% credible intervals from the posterior distribution of coefficients, fitting effects for each of the different parasite groups by using indexing [38]. We ensured convergence across the chains, with $\hat{R}$ values estimated to be 1 for all coefficients. We also compared this model to one that included country as a predictor, but information theoretic measures of support for this more complex model were higher than for the model that only included parasite group, and thus it was not used.

## RESULTS

When comparing the observed parasite species richness to the distribution of predicted values for each country, all of the observed parasite species richness values fell between the 5th and 95th percentiles of the predicted values, indicating that none of the individual observations were exceptionally large or small relative to predictions. However, within a parasite type, results for the different countries tended to follow a consistent pattern.

**Figure 1.**
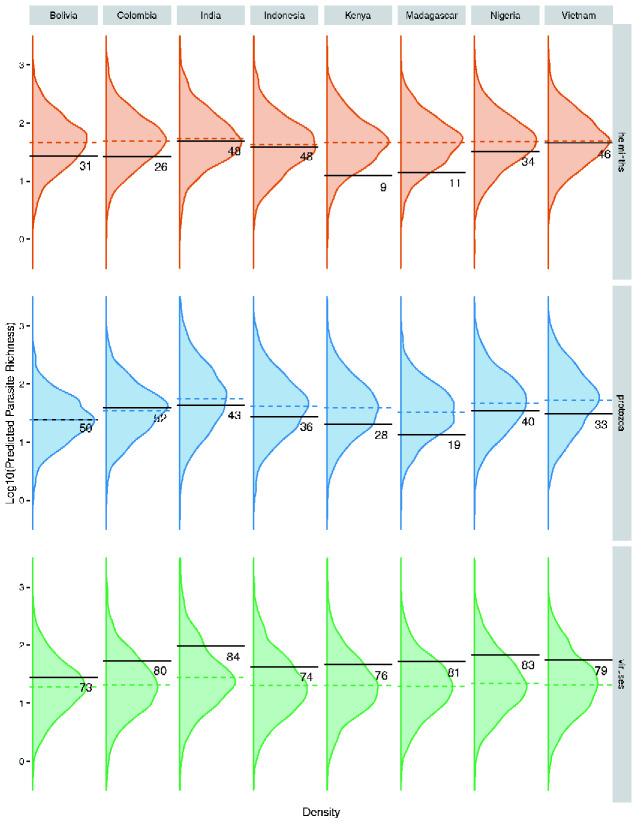
Posterior distributions for the predicted parasite species richness of helminths, protozoa, and viruses for each human population based on models of nonhuman primate parasitism. Colored dotted lines represent the mean of the posterior distributions, and black solid lines represent the observed parasite species richness. The numbers printed below the black lines represent the proportion of predicted values that fall lower than the observed value for parasite species richness.

In analyses of helminth richness, the observed value fell below the mean of the predicted distribution for all eight countries; the mean percentile of the predicted values at which the true value fell across all eight countries was 32%. Kenya and Madagascar had the lowest observed helminth richness relative to the predictions that the model generated, with only 9% and 11% of predictions lower than the observed richness, respectively. Observed richness for India, Indonesia, and Vietnam were close to, but still below, the mean of the predicted distributions (48%, 48%, and 46%, respectively). Thus, all countries had fewer than predicted helminths, though none were extremely divergent from predictions (Fig. 1).

For protozoa, we found a similar pattern, with the observed richness for six countries falling below the mean of the prediction based on patterns in nonhuman primates. For Bolivia, the observed richness was the same as the mean of the prediction, and for Colombia, the observed richness was slightly higher than the mean (52% of the predicted values fall below the observed value). Madagascar was the most extremely under-parasitized country according to the model, with 19% of the predicted values in the posterior distribution being lower than the observed protozoan species richness. Overall, across all eight countries in predictions of protozoan richness, the mean percentile of the posterior distribution at which the true value fell was 38% (Fig. 1).

The results for viruses showed the opposite pattern (Fig. 1). None of the observed richness estimates for the number of viruses in a country was more extreme than the 90% credible interval of the posterior distribution. However, the observed values for all eight countries fell above the mean of the predicted distribution, and the mean percentile of the observed value was 70%. The country with the most extreme observed value relative to the prediction was India, with 84% of predicted values below the true virus species richness, followed closely by Nigeria with 83%.

We then investigated the consistency of results by parasite group using a separate Bayesian model fit (Table 1). This analysis revealed consistently negative deviations from expectations for both helminths and protozoa; that is, observed richness of these groups of parasites was consistently below the mean prediction across the eight countries. In contrast, we found consistently positive deviations for the viruses. Overall, different groups of parasites showed different patterns of change along the human lineage, with support for both of the hypotheses that we investigated, depending on the parasite group.

**Table 1.** Results from MCMC analysis of deviation from phylogenetic predictions (deviation ∼ parasite group, *N*_eff_ = effective sample size across four chains; variance = 0.17)

	Mean	Standard deviation	Lower 5%	Upper 95%	*N*_eff_
Helminths	−0.23	0.06	−0.33	−0.13	10 050
Protozoa	−0.16	0.06	−0.26	−0.06	10 119
Viruses	0.39	0.06	0.29	0.49	10 346

## DISCUSSION

To investigate whether humans are exceptionally parasitized, we compared observed values of parasite species richness in eight human populations to distributions of predicted levels of parasitism based on a nonhuman primate model. No value of observed parasite species richness for a single human population fell outside of the 90% credible interval of the prediction. Our ability to draw conclusions from these individual tests is limited by the wide posterior distributions of our models, which result from a high degree of uncertainty in the underlying model of parasite richness for the 33 primate species in our sample.

We also analyzed the differences between predicted and observed parasite richness across all of these tests, similar to a meta-analysis. In this broader analysis of consistency across populations, we found evidence that human parasite species richness consistently differed from predictions based on patterns in nonhuman primates. The direction of the difference depended on parasite type. For viruses, our findings supported the common view that humans are hyper-parasitized compared to expectations, which is consistent with current thinking about the major epidemiological transitions along the human lineage [1]. For helminths and protozoa, on the other hand, we found evidence consistent with the parasite reduction hypothesis, namely that human hygienic and psychological adaptations may have reduced parasitism for these broad classes of parasites.

The lack of support for these findings in individual countries tempers our conclusions from this broader analysis. Indeed, among all of the primate species for which we predicted parasite species richness, the observed values for human populations were not generally among the most divergent from expectations (see Supplementary Information S2). Until we have statistical models that explain more variation in parasite richness in nonhuman primates, and thus produce tighter posterior predictive distributions for individual countries, we can only cautiously conclude that humans are “hyper” parasitized for viruses, with the magnitude and significance of this effect remaining murky for individual populations. The same caveats apply, in the opposite direction, for protozoa and helminths.

A novel contribution of our approach is to compare the parasites of humans to those of nonhuman primates on an even playing field, in which we control for variables that are known to influence parasitism. Thus, we included individual human populations in geographic locations and range sizes that are comparable to primate species’ geographic ranges, we applied a species richness estimator to the parasite data to adjust for uneven study effort, and we incorporated relevant ecological characteristics into the statistical modeling and resulting predictions.

However, humans remain unique from other primates in many ways that could influence parasite richness itself, or that could affect the discovery of new parasites in humans. For example, country-level populations of humans are not isolated from the globalized population; global travel and trade could contribute to the continued and historical introduction of new parasites into human populations [39] in a way that is not analogous to primate species. For example, local parasite richness of humans is likely to be influenced by immigration of parasites from connected, mobile human populations. This interconnectedness of human populations could be argued to result in higher parasite species richness than would be expected for an isolated population. However, most parasites of nonhuman primates are host-generalists [25], meaning that parasites could immigrate to a host from other host species with overlapping ranges, arguably analogous to the connections between human populations. Thus, we cannot clearly attribute our results to definitional differences in geographic range for human populations and primate species.

Another way in which humans differ from nonhuman primates is that humans receive medical care, and have the ability to report their own symptoms. These factors contribute to higher awareness of a broader diversity of parasite species in humans than in other hosts. Although we applied ecological methods to control for study effort [11] and focused on country-level data, human parasite research—and thus, discovery—may be biased in unique ways that are not fully addressed through the use of these methods. For example, given interests in public health, research has devoted much effort to resolving viral lineages in humans, which could inflate measures of viral richness. Importantly, however, we expect that these biases and many of the other most relevant traits of humans would increase the observed parasite species richness relative to expectations, yet we find evidence of the contrary for two broad parasite groups. Our results thus highlight the importance of testing prominent and even apparently obvious patterns—such as the hyper-parasitism of humans—with statistically rigorous methods.

The differences in results across parasite groups are consistent with previous research identifying taxonomic patterns in the number of host species that a parasite infects [25], which could result in differential probabilities of extinction, emergence, or re-emergence over time [40]. In a comparative study of primate parasites, Pedersen *et al.* [25] found that most helminths were host-specific, viruses tended to be host-generalists, and protozoa were intermediate in their specificity. Specialists may be more prone to extinction than generalists [41], which can potentially use multiple hosts as a sort of “safe haven” when other hosts are declining toward extinction [7]. In addition, despite previous findings that phylogenetically close species should demonstrate the highest degree of host sharing [42, 43], humans have lower similarity of host-specialist parasites with their close relatives, the other great apes, than predicted [4, 7]. In light of these previous findings, our results of lower-than-predicted parasite richness in helminths and protozoa may be the result of a loss of host specialists over evolutionary time. In addition, viruses could be uniquely difficult to extinguish, independent of host specificity, because of their faster rates of evolution and greater potential to shift between hosts [44, 45]. These factors could explain the consistently higher observed than predicted richness of viruses in humans.

Another explanation for the taxonomic patterns of observed relative to predicted richness is that they reflect selection on parasites with different transmission modes. For example, the complex and environmental life cycles that are more common among helminths and protozoa might make them more susceptible than viruses to extinction as a result of hygienic interventions like toilets [46]. Humans’ urban ecosystem may also reduce their exposure to parasites with complex lifestyles, as has been shown for nonhuman primates that live in urban habitats [47]. In contrast, more of the viruses can be transmitted via close contact [25]. In fact, many viral, close-contact transmitted diseases emerged once humans lived in high enough densities to sustain them (i.e., “crowd epidemic diseases”), and could not have persisted in small, dispersed hunter-gatherer groups [3, 48]. In addition to the nearly global human distribution, humans are expanding their range on a more local scale into forests and wild areas, providing more opportunities for transmission from other species [49]. As noted above, our results may also reflect research biases that our methods could not fully account for, such as greater effort to resolve viral diversity in humans relative to nonhuman primates.

Therefore, for a number of reasons, viral diseases may be more likely to emerge and harder to eliminate via many of the mechanisms that could have extinguished helminths and protozoa in the course of human evolution. Given this perspective, it is not surprising that viruses are so numerous among the novel infectious diseases that have emerged in the past 40 years, including coronaviruses, Ebola virus, Zika virus, and West Nile virus [50].

## CONCLUSIONS AND IMPLICATIONS

Overall, the results presented here provide evidence for both the parasite reduction and hyperparasitism hypotheses, with the former supported by patterns in helminths and protozoa, and the latter in viruses. Although caution is needed, given that differences from expectations were only found in the consistency tests across all countries, these findings suggest that human epidemiological transitions and cultural adaptations may have differentially impacted the extinction and emergence of parasites across human evolutionary history, depending on the biology of those parasites. These results raise many questions for future research, including how patterns of parasitism vary across human populations with differing lifestyles and cultural practices on a finer scale. For example, the human populations in this study comprise a wide range of urban and rural settings, access to plumbing, refrigeration, and sanitation resources, and cultural beliefs, all of which may influence parasite richness. Understanding how these factors shape parasite richness within individual human populations will provide clearer explanations for the broad-scale patterns that we uncover here.

## Supplementary Data

Supplementary data is available at *EMPH* online.

## Supplementary Material

eoab009_Supplementary_Data
