# Supplementary material for: Epidemiological transitions in human evolution and the richness of viruses, helminths, and protozoa
Source: Evol Med Public Health. 2021 Feb 23;9(1):139–48. doi: 10.1093/emph/eoab009 (PMC7953414; doi:10.1093/emph/eoab009)
Supplement: eoab009_Supplementary_Data [file eoab009_supplementary_data.zip › R1_Supplementary Information_S1_Bayesian Model Summary.docx]

**Supplementary Information – S1**: Results of Bayesian MCMC phylogenetic least-squares regression model fitting. We ran 2,000 iterations for each parasite type, for each of 8 human countries, and averaged the estimates across all of these runs; thus, the values presented in the tables below each represent the mean and standard deviation of coefficient estimates from 16,000 runs. The probability of a variable being included in the model represents the proportion of runs in which the MCMC algorithm included the variable of interest (i.e. the coefficient estimate was ≠ 0). The ‘overall mean coefficient estimate’ includes zeros for the runs when the variable was excluded from the model, while the ‘mean coefficient in models that include the variable’ excludes those zeros, and calculates the mean only from the runs that included the variable. For example, in Supplementary Table S1, for helminths, a non-zero coefficient for Log of Population Density was included in 12.7% of models. The mean coefficient estimate in 16,000 models was 0.006. In the 2,032 (12.7% of 16,000) models that included an estimate for Log of Population Density, the mean coefficient was 0.05.

Supplementary Table S1. Helminth model summary statistics. Mean $\lambda$ = 0.539, SD = 0.292.

| Variable | Probability of Inclusion in Model | Overall Mean Coefficient Estimate | Overall SD of Coefficient Estimate | Mean Coefficient in Models that Include Variable | SD of Coefficient in Models that Include Variable |
| --- | --- | --- | --- | --- | --- |
| Intercept | 1 | 1.188 | 0.651 | 1.188 | 0.651 |
| LogPopDens | 0.127 | 0.006 | 0.050 | 0.050 | 0.133 |
| LogBodyMass | 0.236 | 0.037 | 0.146 | 0.157 | 0.267 |
| LogGeoRange | 0.224 | 0.001 | 0.119 | 0.006 | 0.252 |
| LogLatRange | 0.464 | 0.197 | 0.317 | 0.424 | 0.347 |

Supplementary Table S2. Protozoa model summary statistics. Mean $\lambda$ = 0.614, SD = 0.273.

| Variable | Probability of Inclusion in Model | Overall Mean Coefficient Estimate | Overall SD of Coefficient Estimate | Mean Coefficient in Models that Include Variable | SD of Coefficient in Models that Include Variable |
| --- | --- | --- | --- | --- | --- |
| Intercept | 1 | 0.886 | 0.523 | 0.886 | 0.523 |
| LogPopDens | 0.708 | 0.223 | 0.181 | 0.315 | 0.131 |
| LogBodyMass | 0.315 | 0.087 | 0.209 | 0.277 | 0.293 |
| LogGeoRange | 0.127 | -0.006 | 0.073 | -0.050 | 0.199 |
| LogLatRange | 0.206 | 0.018 | 0.156 | 0.087 | 0.335 |

Supplementary Table S3. Virus model summary statistics. Mean $\lambda$ = 0.392, SD = 0.215.

| Variable | Probability of Inclusion in Model | Overall Mean Coefficient Estimate | Overall SD of Coefficient Estimate | Mean Coefficient in Models that Include Variable | SD of Coefficient in Models that Include Variable |
| --- | --- | --- | --- | --- | --- |
| Intercept | 1 | 0.360 | 0.747 | 0.360 | 0.747 |
| LogPopDens | 0.323 | 0.071 | 0.134 | 0.220 | 0.150 |
| LogBodyMass | 0.337 | 0.095 | 0.207 | 0.283 | 0.272 |
| LogGeoRange | 0.253 | 0.031 | 0.133 | 0.124 | 0.242 |
| LogLatRange | 0.472 | 0.192 | 0.326 | 0.407 | 0.372 |
