# Supplementary material for: Epidemiological transitions in human evolution and the richness of viruses, helminths, and protozoa
Source: Evol Med Public Health. 2021 Feb 23;9(1):139–48. doi: 10.1093/emph/eoab009 (PMC7953414; doi:10.1093/emph/eoab009)
Supplement: eoab009_Supplementary_Data [file eoab009_supplementary_data.zip › R1_Supplementary Information_S2_Non-Human Primate Species Predictions.docx]

Figure S1: Observed vs. predicted parasite species richness of helminths for primate host species in the sample. The histogram represents the distribution of predicted values, with log_10_-transformed parasite species richness on the x-axis. The vertical dotted gray line represents the mean of the predicted distribution, and the black line represents the observed value.

Figure S2: Observed vs. predicted parasite species richness of protozoa for primate host species in the sample. The histogram represents the distribution of predicted values, with log_10_-transformed parasite species richness on the x-axis. The vertical dotted gray line represents the mean of the predicted distribution, and the black line represents the observed value.

Figure S3: Observed vs. predicted parasite species richness of viruses for primate host species in the sample. The histogram represents the distribution of predicted values, with log_10_-transformed parasite species richness on the x-axis. The vertical dotted gray line represents the mean of the predicted distribution, and the black line represents the observed value.
